# Editorial: Pathophysiology of the Basal Ganglia and Movement Disorders: Gaining New Insights from Modeling and Experimentation, to Influence the Clinic

**DOI:** 10.3389/fnhum.2017.00466

**Published:** 2017-09-20

**Authors:** Daniela S. Andres, Marcelo Merello, Olivier Darbin

**Affiliations:** ^1^Laboratory of Neuroengineering, Science and Technology School, National University of San Martín San Martín, Argentina; ^2^Movement Disorders Section, Neuroscience Department, Raul Carrea Institute for Neurological Research, Fundación para la Lucha contra las Enfermedades Neurológicas de la Infancia (FLENI) Buenos Aires, Argentina; ^3^Department of Neurology, University South Alabama Mobile, AL, United States; ^4^Division of System Neurophysiology, National Institute for Physiological Sciences Okazaki, Japan

**Keywords:** Parkinson's disease, movement disorders, Huntington's disease, basal ganglia, temporal structure, personalized therapies, alpha synuclein (α syn), non-motor symptoms

The human brain is complex at every level, from the scale of single neurons to microcircuits and large neuronal networks. Although this complexity is well known and studied in neuroscience, few tools or concepts of complex analysis have been transferred to the clinic yet. In the case of the basal ganglia, there has been much debate about the necessity to include nonlinear concepts into pathophysiology models (Andres and Darbin, in press). However, for new approaches to make an impact on the clinic active research is needed on many fronts: new clinical, experimental and modeling insights are crucial. In other words, research in the field of basal ganglia and related disorders is becoming increasingly interdisciplinary. On this research topic different authors challenge classic paradigms of basal ganglia pathophysiology and movement disorders in 6 areas of research. Main findings and breakthroughs are summarized in the next paragraphs.

## Basal ganglia models and theory

- Current theories of the basal ganglia are not always consistent with clinical and experimental observations. New models need to be built based on advances in the fields of complexity, chaos and non-linear systems (Montgomery).
- A new model emphasizes the role of cognition in motor control, showing that freezing of gait can result from increased risk sensitivity in patients with Parkinson's disease (PD) (Muralidharan et al.).
- Action selection concepts and pharmacokinetics combined in a mixed modeling approach can be used to study how dopamine affects a motor task at different stages of PD (Baston et al.).

## Basal ganglia functions

- The basal ganglia together with the cerebellum and prefrontal cortical areas play a role in time processing, which is altered in movement disorders and affects both motor and cognitive performance (Avanzino et al.).
- Dopamine signaling influences the level of physical activity. Chronic exposure to obesogenic diets cause striatal dopamine dysfunction, which might be related to the difficulty of people with obesity to increase their physical activity (Kravitz et al.).
- Local field potentials (LFP) observations in patients with PD show that the subthalamic nuclei (STNs) are bilaterally involved in voluntary muscle contraction and relaxation, with characteristic activity in the theta (4–7 Hz), beta (14–35 Hz), and gamma bands (40–100 Hz) (Kato et al.).

## Molecular pathways and neurotransmission

- Accumulation of alpha-synuclein (αSyn) in Lewy bodies and Lewy neurites characterizes the progression of Parkinson's disease. The discovery of cell-to-cell propagation of αSyn opens new therapeutic avenues for the treatment of PD and related disorders (Prymaczok et al.).
- Huntington's disease (HD) is considered as a paradigm of epigenetic dysregulation. Cell-type specific techniques and 3D-based methods can be used to advance knowledge in the context of brain region vulnerability in neuordegenerative diseases, leading to the design of new therapeutic targets (Francelle et al.)
- Dysregulation of glutamate in the corticostriatal pathway is implicated in HD. Alterations of dopamine, a modulator of glutamatergic activation, also plays a role in deficits of neuronal communication throughout the basal ganglia in HD (Bunner and Rebec).

## Non-motor symptoms of basal ganglia disorders

- Apathy is a cardinal symptom of PD, but its pathophysiology is poorly understood. A new animal model (VMAT2 deficient mice) shows an apathetic-like phenotype that might be independent of depressive-like symptoms. This is a step forward to study the biological substrates of apathy in PD. (Baumann et al.)
- A study based on electroencephalograms (EEG) of PD patients and aged-matched healthy individuals shows that pharmacologic treatment helps maintaining long-term action-outcome representations in PD patients, but not the initial experience of action-effect (Bednark et al.).

## Signal analysis and clinical approaches

- The temporal structure function is a robust and simple to compute tool for the analysis of neuronal activity, which helps identifying random, oscillatory and non-linear behavior in the dynamics of single neurons. This technique can be used to quantify complex neuronal activity in healthy and PD neurons (Nanni and Andres).
- A new cost-effective screening protocol for parkinsonism based on combined objective and subjective monitoring of balance using a game industry balance board might be a strategy for PD screening in communities with limited access to healthcare (Darbin et al.).
- Bicycling ability remains preserved in PD patients who suffer freezing of gait, but the neural mechanisms underlying this observation are not known. A new experimental setup allows to investigate this phenomenon, combining recording of basal ganglia LFP and scalp EEG in PD patients while bicycling, walking or performing other motor tasks (Gratkowski et al.).
- Disparate patterns of subcortical degeneration evidenced by automated volumetric magnetic resonance imaging can explain some differences in symptoms between PD clinical subtypes, such as gait disturbances and cognitive functions. This finding may help to design personalized therapeutic approaches in the future (Rosenberg-Katz et al.).
- Quantification of specific functional deficits of gait could provide a basis for locating the source and extent of neurological damage in PD, aiding clinical decision-making for individualizing therapies (König et al.).

## Deep brain stimulation (DBS)

- A new method uses intraoperative stimulation test data to identify optimal implant position of DBS leads by relating electric field simulations to patient/specific anatomy and the clinical effects of stimulation as measured by accelerometry (Hemm et al.).
- A new study based on near-infrared spectroscopy (NIRS) in PD patients concludes that therapeutic DBS promotes neuronal network remodeling in the prefrontal cortex (Morishita et al.).
- Impulsivity is related to an abnormally fast reaction time in high conflict situations, which is high under DBS of the STN. In a computational model, reaction time can be controlled varying the DBS electrode position within the STN and causing antidromic activation of the globus pallidus externa (GPe) (Mandali and Chakravarthy).

The results published in this topic promise great advancements in coming years in the field of basal ganglia pathophysiology and related disorders.

## Author contributions

DA, MM, and OD are responsible for the full content of this article.

### Conflict of interest statement

The authors declare that the research was conducted in the absence of any commercial or financial relationships that could be construed as a potential conflict of interest.
